# Mitochonic Acid 5 Increases Ram Sperm Quality by Improving Mitochondrial Function during Storage at 4 °C

**DOI:** 10.3390/ani14030368

**Published:** 2024-01-23

**Authors:** Ruyuan Wang, Luwei Liu, Lingjiang Min, Adedeji O. Adetunji, Xin Kou, Kaifeng Zhou, Zhendong Zhu

**Affiliations:** 1 College of Animal Science and Technology, Qingdao Agricultural University, No. 700 Changcheng Road, Qingdao 266109, China; 2 Department of Agriculture, University of Arkansas at Pine Bluff, Pine Bluff, AR 71601, USA; 3 Hongde Livestock Farm, Yingli Town, Shuoguang 262717, China; 4 Shandong Provincial Animal Husbandry General Station, Jinan 250022, China

**Keywords:** liquid storage, mitochondria, mitochonic acid 5, ram sperm

## Abstract

**Simple Summary:**

This study investigated the efficacy of mitochonic acid 5 (MA-5) supplementations in enhancing ram sperm quality during 4 °C storage. Cold shocks, osmotic stresses, and oxidative stresses during storage can lead to irreparable damage. Ram sperm diluted with a tris-citrate-glucose extender containing varied MA-5 concentrations were stored for different durations. Notably, the addition of MA-5, particularly at 10 nM, significantly improved sperm quality by enhancing motility, membrane integrity, and acrosomal integrity. MA-5 also increased mitochondrial membrane potential, reduced reactive oxygen species (ROS) levels, and elevated adenosine triphosphate (ATP) levels in ram sperm. The findings suggest that MA-5 preserves high mitochondrial function during liquid storage, providing a promising avenue for further research in ram sperm preservation and specific proteins.

**Abstract:**

Semen preservation involves lengthening sperm’s fertile lifespan without any detrimental effects on its biochemical, functional, and ultrastructural properties. Liquid storage at 4 °C is a ram sperm preservation method. However, this method of storage causes irreversible damage due to cold shocks, osmotic stresses, oxidative stresses, and reductions in sperm metabolism. The present study aims to investigate whether the supplementation of mitochonic acid 5 (MA-5) in a sperm extender could improve chilled ram sperm quality and elucidate its mechanism of action. Ram sperm were diluted with a tris-citrate-glucose extender containing different concentrations of MA-5 (0, 0.1, 1, 10, and 100 nM) and stored at 4 °C for up to 48 h. Sperm motility, membrane integrity, acrosome integrity, mitochondrial membrane potential, reactive oxygen species (ROS) level, ATP content, and the expression of NADPH dehydrogenase subunits 1 (MT-ND1) and NADPH dehydrogenase subunits 6 (MT-ND6) were evaluated. It was observed that compared to the control, the 10 nM MA-5 treatment significantly (*p* < 0.05) increased total motility (82 ± 3.5% vs. 76 ± 5.9%), progressive motility (67.6 ± 8.2% vs. 51 ± 8.3%), and other parameters (straight-line velocity (VSL), average path velocity (VAP), and curvilinear velocity (VCL)). In addition, 10 nM MA-5 supplementation also improved ram sperm membrane integrity and acrosomal integrity as well increased mitochondrial membrane potential (51.1 ± 0.7% vs. 37.7 ± 1.3%), reduced ROS levels, and elevated adenosine triphosphate (ATP) contents. Furthermore, a Western blot analysis demonstrated that the addition of MA-5 significantly (*p* < 0.05) increased the expression of MT-ND1 and MT-ND6 proteins in ram sperm, with the 10 nM MA-5 treatment resulting in the highest expression level. These results suggest that MA-5 improves ram sperm quality by maintaining high sperm mitochondrial function during liquid storage at 4 °C.

## 1. Introduction

Semen preservation is a method to prolong the fertile life of sperm that plays a key role in artificial insemination [1]. This process includes storing sperm in either a liquid or cryopreserved state [2]. Slowing down key biochemical functions is crucial in the liquid preservation of ram sperm. To achieve this, sperm are kept in cooled or chilling conditions within a range of 0–15 °C [3]. However, the preservation of both chilled or fresh ram sperm has been limited to 24 h, with an average decline of 10–35% in fertility per day during cervical insemination [1]. As a result, the distance between the sire’s location and the place of insemination has been constrained by this time limitation [1]. Additionally, during the liquid storage process at 4 °C, sperm experience irreversible damage due to cold shocks [4,5], osmotic stresses [5,6,7], oxidative stresses [6], and a decrease in sperm metabolism [8]. A significant challenge encountered in the preservation of ram sperm is the accumulation of reactive oxygen species (ROS) [9], which cause oxidative damage to sperm during prolonged liquid storage at 4 °C, resulting in damage to the plasma membrane [10]. Additionally, heightened ROS levels were associated with impaired ATP production and damage to the mitochondrial transcription system in sperm [11]. 

Reactive oxygen species (ROS) are generated through mitochondrial activity during the oxidative phosphorylation (OXPHOS) process that occurs when there is a reduced cell oxygen content [11]. While this process may be beneficial, elevated levels of ROS are detrimental to sperm function as they lead to oxidative stress due to their unique physical architecture and biochemical composition [12,13]. Oxidative stress decreases sperm function [8]; thus, reducing the ROS level will help lengthen sperm’s fertile lifespan. 

Mitochondria exhibit a compacted matrix with a condensed morphology in mature sperm, a structural configuration designed to enhance energy production efficiency. It is important to note that species variations contributed to differences in this mitochondrial organization. Additionally, mitochondria feature a specialized membrane structure, with the inner mitochondrial membrane identified as the site for ATP production [14]. These intricate mitochondrial characteristics underscore the nuanced and species-specific aspects of sperm biology, particularly in the realm of energy production and cellular function, as revealed in past research. For example, the structure provided a foundation for lactate/pyruvate, aspartate/malate, and glycerol-3-phosphate [15]. Additionally, sperm mitochondria were found to contain at least three distinct shuttles for the transport of reducing equivalents into the mitochondrial matrix, facilitating the energy production necessary for sperm function [16]. It is worth noting that the inner mitochondrial membrane plays a crucial role in controlling metabolite transporters and the oxidative status of the cell through oxidative phosphorylation and ion transport [17]. Moreover, mitochonic acid-5 (MA-5), a mitochondria-targeting chemical isolated from plants [18], was found to play a key role in the regulation of cellular mitochondrial function [19].

Mitochonic acid-5 enhanced mitochondrial function by reducing mitochondrial oxidative stress and accelerating mitochondrial energy metabolism in fibroblasts [19]. Moreover, previous studies revealed that MA-5 decreased mitochondrial diseases by increasing the ATP content in fibroblasts and B cells [19,20]. MA-5 was found to upregulate cardiac and renal respiration in a mitochondrial disease model to increase survival chances in renal tubules and cardiac myocytes [21]. However, information about the effect of MA-5 on sperm is limited; thus, in the present study, we hypothesized that the addition of MA-5 to a sperm extender may improve ram sperm quality by stimulating mitochondrial function during liquid storage at 4 °C for up to 48 h. Understanding the mechanism through which MA-5 enhances mitochondrial function and sperm quality control can help in developing interventions for the treatment of male infertility. 

## 2. Materials and Methods

### 2.1. Chemicals

Routine chemicals and reagents were purchased from Sigma-Aldrich^®^ Agricultural Technology Development Co., Ltd. (Shanghai, China). Mitochonic acid 5 (cat. no. S0881), 4-(2,4-difluorophenyl)-2-(1*H*-indole-3-yl)-4-oxobutanoic acid, was purchased from SelleckChem.

### 2.2. Ethical Approval 

All animals and experimental procedures were approved by the Qingdao Agriculture University Institutional Animal Care and Use Committee (QAU1121010).

### 2.3. Ram Semen Collection and Processing

Semen was collected from six fertile and healthy rams aged about 2 years using an artificial vagina, with three consecutive collections conducted within a week. The semen was collected within three weeks. The rams were individually housed and received carefully curated diets and exclusive access to water at Hongde Livestock Farm in ShuoGuang city. A total of 54 ejaculates were procured from these six rams, each maintained separately during transit to the laboratory. The assessment of ejaculated semen motility employed a computer-assisted sperm analysis (CASA) system, imposing a rigorous inclusion criterion demanding over 90% motility in the semen samples [22]. To minimize individual variations, semen from the six rams were judiciously pooled, partitioned into five aliquots, diluted with a tris-citrate-glucose (TCG) extender containing different concentrations of MA-5 (0, 0.1, 1, 10, and 100 nM), and stored at 4 °C for up to 48 h. The TCG extender was composed of 250 mM of Tris, 83 mM of trisodium citrate, and 69 mM of glucose.

### 2.4. Evaluation of Sperm Motility Using a Computer-Assisted Sperm Analysis (CASA) System

According to Zhu et al. (2019) [11], 5 μL of each sperm sample was dispensed into a pre-warmed Makler sperm-counting chamber (10 µm depth; Haifa Instruments, Haifa, Israel). Sperm trajectories (0.5 s, 45 frames) were systematically captured at 60 Hz, employing a computer-assisted sperm analysis (CASA) system (HT CASA-Ceros II; Hamilton, MA, USA). More than 200 individual trajectories were meticulously recorded. The recorded parameters were straight-line velocity (VSL, μm/s), curvilinear velocity (VCL, μm/s), average path velocity (VAP, μm/s), linearity (LIN, %), straightness (STR, %), wobble (WOB %), total motility (%), progressive motility (%), and non-progressive motility (%). VCL, VSL, STR, LIN, VAP, and WOB were only calculated for motile sperm.

### 2.5. Evaluation of Sperm Membrane Integrity and Acrosome Integrity

SYBR-14 (Sigma-Aldrich, Shanghai, China) was used in combination with propidium iodide (PI) to evaluate the membrane integrity of the sperm [23]. In this study, a 100 µL sperm sample was first mixed with 0.1 µL of SYBR-14 and 0.5 µL of PI. It was then incubated in the dark at 37 °C for 10 min. Subsequently, the stained sperm were evaluated using a fluorescence microscope (ZEISS DM200LED, Oberkochen, Germany), as shown in Supplementary Figure S1A. Sperm with bright green fluorescence were considered intact (white arrow), while sperm with red fluorescence were indicated to have plasma membrane damage (red arrow). During the final analysis, a minimum of 200 sperm were examined and individually scored on each slide. The evaluation of sperm acrosome integrity was conducted using fluorescence isothiocyanate peanut agglutinin (FITC-PNA) and PI [23]. First, 30 µL of sperm was placed on a slide and fixed in 75% methanol. The fixed sperm sample was then incubated with 30 μL of an FITC-PNA solution in 37 °C darkness for 30 min and then incubated with another 20 μL of PI solution for 10 min. Acrosome integrity was detected using a fluorescence microscope (ZEISS DM200LED), and it was ensured that at least 200 sperm were examined per slide. As shown in Supplementary Figure S1B, sperm showing bright green fluorescence were considered acrosome-intact (white arrows), while sperm with no green or speckled green fluorescence were indicated to have acrosome damage (red arrows). 

### 2.6. Mitochondrial Activity

A JC-1 Assay Kit (911, InMunoChemistry Technologies, LLC, Davis, CA, USA) was used to measure sperm mitochondrial activity [24]. Briefly, sperm samples were incubated with 500 μL of 1× working solution at 37 °C for 30 min in the dark. Then, mitochondrial activity was analyzed via flow cytometry using a filter with a bandwidth of 574/26 nm (AttuneOR NxT Acoustic Focusing Cytometer, FACSCalibur, BD Biosciences, Carlsbad, CA, USA) and measured as the mean fluorescence intensity (MFI) of JC-1 orange aggregates. A total of 50,000 sperm events were analyzed.

### 2.7. Sperm Mitochondrial ROS Level

The MitoSOX™ Red Assay Kit (M36008, Thermo Fisher Scientific K.K, Shangai, China.) was employed for the analysis of intracellular sperm mitochondrial ROS generation [24], following the manufacturer’s instructions. Sperm samples were incubated with 500 μL of MitoSOX™ Red reagent working solution (5 μM) at 37 °C for 10 min in the dark. Subsequently, mitochondrial ROS levels were assessed by flow cytometry using a filter with a bandwidth of 574/26 nm, and the measurement was expressed as the mean fluorescence intensity (MFI). A total of 20,000 sperm-specific events were analyzed.

### 2.8. Sperm ATP Contents

Sperm ATP contents were evaluated utilizing an Enzylight™ ATP Assay Kit (EATP-100, Bioassay System, Hayward, CA, USA) [25]. In brief, sperm samples were combined with the assay buffer and substrates, followed by a luminescence measurement using a luminometer (2030 Multilabel Reader ARVO X4; PerkinElmer Inc., Waltham, MA, USA).

### 2.9. Western Blotting

According to the previous study [24], total protein extraction from sperm was carried out in a sodium dodecyl sulfate (SDS) sample buffer. Following this, protein separation was performed using 12.5% SDS-polyacrylamide gel electrophoresis (SDS-PAGE), and the separated proteins were transferred to a polyvinylidene difluoride (PVDF) blotting membrane (GE Bioscience, Newark, NJ, USA). To mitigate nonspecific binding, the membranes underwent incubation in Tris-buffered saline (TBS) containing 0.1% (*v*/*v*) Tween-20 and 5% (*w*/*v*) bovine serum albumin (Life Technologies, Grand Island, NY, USA). Primary antibodies, including anti-mitochondrial NADPH dehydrogenase subunit 1 (anti-MT-ND1; 1973-1-AP, Proteintech, Rosemont, IL, USA), anti-mitochondrial NADPH dehydrogenase subunit 6 (anti-MT-ND6; bs-3955R; Bioss, Inc., Boston, MA, USA), and anti-α-tubulin (2148; Cell Signaling Technology, Inc., Danvers, MA, USA), were diluted to 1:1000 in 5% bovine serum albumin in TBS-Tween and applied overnight at 4 °C. Following primary antibody incubation, HRP-conjugated secondary antibodies (goat anti-rabbit for MT-ND1, MT-ND6, and α-tubulin) were applied at a 1:5000 dilution. After rigorous washing in TBST, enhanced chemiluminescence (ECL) detection was performed using the ECL system per the manufacturer’s specifications (ED0016-B, Sparkjade, Jinan, China). The blots were exposed to Fuji X-ray film (Fujifilm, Tokyo, Japan). Subsequently, band intensities were quantified using a Gel-Pro Analyzer (Media Cybernetics, Rockville, MD, USA).

### 2.10. Statistical Analysis

All data underwent rigorous assessments for normality and homogeneity of variance before a statistical analysis was conducted. Statistical comparisons across three replicates were executed using a one-way analysis of variance, followed by Duncan’s new multiple range test (95% confidence interval). The results are presented as mean ± standard deviation (SD) values. Treatments were considered statistically distinguishable from one another when the significance level was set at *p* < 0.05.

## 3. Results

### 3.1. Effect of Mitochonic Acid 5 on Sperm Motility Parameters

**Figure 1:**
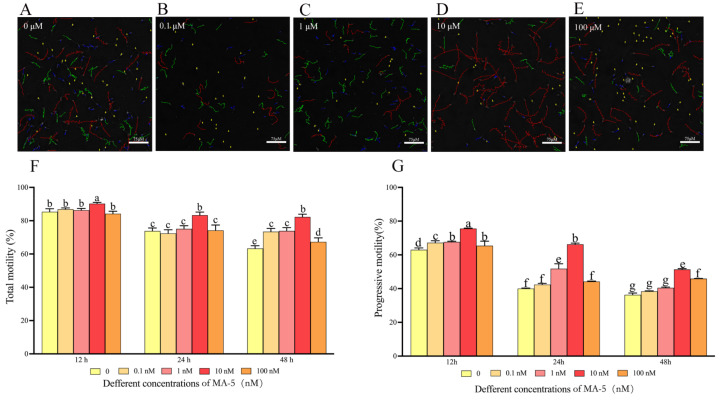
MA-5 increased ram sperm viliablity in liquid storage at 4 °C. (**A**–**E**) A computer-aided sperm analysis (CASA) was utilized to determine sperm viability following a 48 h incubation with or without MA-5 (*n* = 3). In panels (**F**,**G**), total motility and progression motility were assessed in ram sperm after 12 to 48 h of incubation with MA-5 at concentrations ranging from 0 to 100 nM (*n* = 6). Significant differences (*p* < 0.05), denoted by different letters within a line, were identified using a one-way ANOVA and Dunnett’s test, comparing samples to the medium without MA-5.

As shown in Figure 1A–E, the sperm tracks generated from the CASA system revealed that the addition of 10 nM MA-5 significantly (*p* < 0.05) improved ram sperm motility patterns after 48 h of storage. Indeed, the data generated from CASA showed that the value of ram sperm motility in the 10 nM MA-5 treatment was higher than the control at 12 h, 24 h, and 48 h time points at 4 °C, while the other treatments were not different. Interestingly, compared to the control, the addition of MA-5 significantly increased ram sperm progressive motility during 12 h of storage, with the 10 nM MA-5 treatment having the highest value for progressive motility at 12 h, 24 h, and 48 h time points (Figure 1F,G). In Table 1, supplementation of 10 nM MA-5 to the extender significantly increased the VSL, VCL, VAP, LIN, STR, and WOB parameters. In addition, the 10 nM MA-5 treatment had the lowest value for no progression among other treatments. 

### 3.2. Mitochonic Acid 5 Affected Ram Sperm Membrane Integrity and Acrosome Integrity 

**Figure 2:**
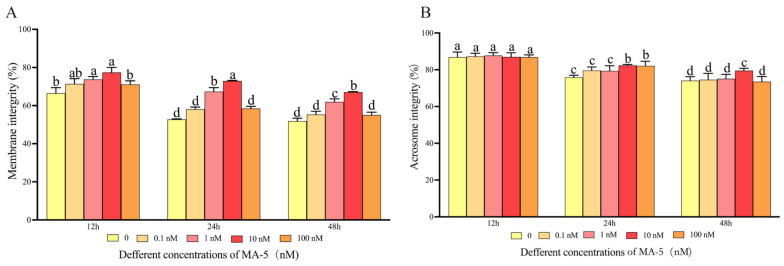
Effect of MA-5 on the morphology of ram sperm in liquid storage at 4 °C. (**A**) Assessment of sperm cell membrane integrity following 12–48 h of incubation with MA-5 at concentrations ranging from 0 to 100 nM (*n* = 3). Significance, denoted by different letters within a line, indicates a notable difference (*p* < 0.05) as determined by a one-way ANOVA and Dunnett’s test, relative to the medium without MA-5. (**B**) Evaluation of sperm cell acrosome integrity after 12–48 h incubation with MA-5 at concentrations ranging from 0 to 100 nM (*n* = 3). Significance, indicated by distinct letters within a line, denotes a noteworthy difference (*p* < 0.05) determined through a one-way ANOVA and Dunnett’s test, in comparison to the medium without MA-5.

As shown in Figure 2A, ram sperm membrane integrity was significantly (*p* < 0.05) decreased during storage at 4 °C. The addition of 1 nM or 10 nM MA-5 to the diluted medium significantly improved (*p* < 0.05) ram sperm membrane integrity compared to the control. However, the 0.1 nM and 100 nM treatments were not different from the control for all time points. Moreover, the 10 nM MA-5 treatment had the best effect on ram sperm membrane integrity among the treatments for all time points (Figure 2A, *p* < 0.05). Additionally, in terms of the sperm being acrosome-intact, ram sperm acrosome integrity decreased during the period of 48 h of storage, and the addition of 10 nM MA-5 to the medium significantly (Figure 2B, *p* < 0.05) increased the number of ram sperm with acrosome integrity at 24 h and 48 h points of storage. However, the percentage of acrosome-intact sperm presented no significantly difference (*p* < 0.05) among all the treatments at 12 h of storage. 

### 3.3. Mitochonic Acid 5 Increased Sperm Mitochondrial Membrane Potential and ATP Content, Decreasing the ROS Level

**Figure 3:**
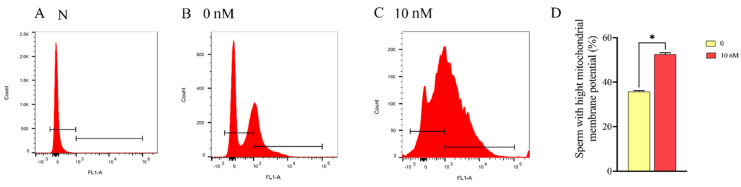
Effect of MA-5 on mitochondrial membrane potential in liquid storage at 4 °C. (**A**) negative control (N);(**B**) 0 nM MA-5 treatment; (**C**) 10 nM MA-5 treatment; (**D**) evaluation of MA-5’s impact at diverse concentrations (0 and 10 nM) on ram sperm exhibiting high mitochondrial membrane potential (*n* = 3). Values are specified as means ± standard deviation (SDs). * *p* < 0.05 (data from three replicates were compared using either Student’s *t*-test or a one-way analysis of variance followed by Dunnett’s test).

**Figure 4:**
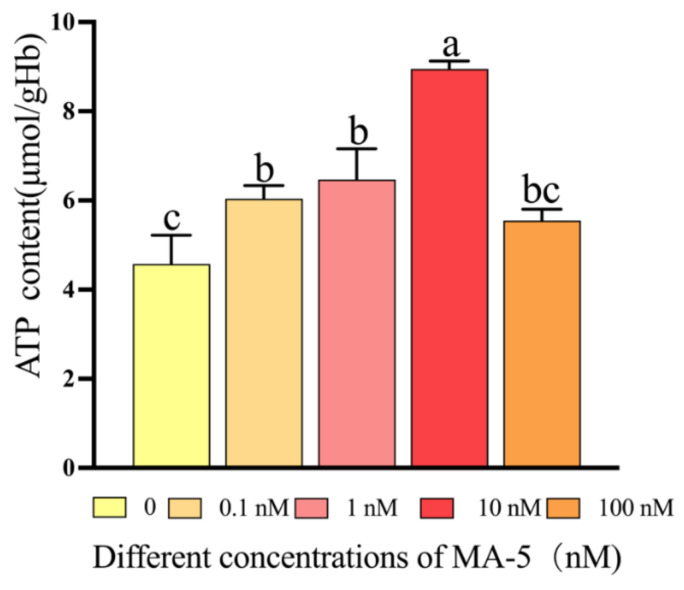
Effect of MA-5 on ATP content in liquid storage at 4 °C. Intracellular ATP contents were measured in ram sperm cells exposed to 0.1% DMSO, 0 nM, 0.1 nM, 1 nM, 10 nM, and 100 nM MA-5 (*n* = 3). Values are specified as means ± standard deviation (SDs). Different letters within a line indicate significant difference (*p* < 0.05); one-way ANOVA and Dunnett’s test vs. sample without MA-5 treatment.

**Figure 5:**
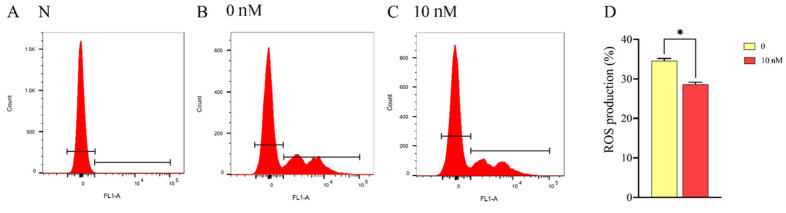
Effect of MA-5 on ROS level in liquid storage at 4 °C (**A**–**D**). (**A**) Negative control (N); (**B**) 0 nM MA-5 treatment; (**C**) 10 nM MA-5 treatment; (**D**) quantification of mitochondrial ROS production in ram sperm mitochondria treated with MA-5 (*n* = 3). Values are specified as means ± standard deviations (SDs). * *p* < 0.05 (data from three replicates were compared using either Student’s *t*-test or a one-way analysis of variance followed by Dunnett’s test).

Sperm mitochondrial membrane potential is important for sperm function. The ram sperm mitochondrial membrane potential in the control and 10 nM MA-5 treatment were analyzed after 48 h of preservation at 4 °C. As depicted in Figure 3A–D, the addition of 10 nM MA-5 to the diluted medium significantly (*p* < 0.05) increased the ram sperm mitochondrial membrane potential compared to the control (Figure 3D, *p* < 0.05). Interestingly, when the ram sperm ATP contents were analyzed after 48 h of preservation, it was observed that addition of MA-5 from 0.1 nM to 10 nM significantly increased the ATP content (Figure 4, *p* < 0.05), and the lowest ROS levels were observed for the 10 nM MA-5 treatment. Notably, the supplementation of 10 nM MA-5 significantly reduced ROS levels compared to the control (Figure 5A–D, *p* < 0.05). 

### 3.4. Mitochonic Acid 5 Increased the Expression of MT-ND1 and MT-ND6 Proteins in Ram Sperm after 48 h of Preservation

**Figure 6:**
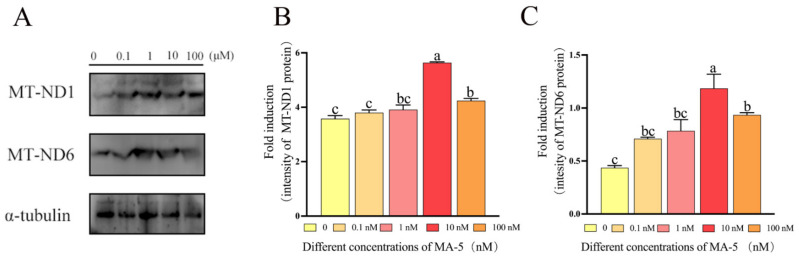
Effect of MA-5 on mitochondrial energy in liquid storage at 4 °C. (**A**) Western blot analysis of MT-ND1 and MT-ND6 in ram sperm. (**B**) Impact of MA-5 on MT-ND1 activity in ram sperm. Values are presented as means ± standard deviations (SDs). The mitochondrial production of proteins with MA-5 and its effect on mitochondrial function were assessed (*n* = 3). Different letters within a line denote significant differences (*p* < 0.05), determined through a one-way ANOVA and Dunnett’s test, relative to the medium without MA-5. (**C**) The impact of MA-5 on MT-ND6 activity in ram sperm. Values are specified as means ± standard deviations (SDs). The mitochondrial production of proteins with MA-5 and its effect on mitochondrial function were evaluated (*n* = 3). Different letters within a line signify significant differences (*p* < 0.05), as determined by a one-way ANOVA and Dunnett’s test, compared to the medium without MA-5.

Utilizing the Western blot method, we employed specific antibodies to detect the presence of MT-ND1 and MT-ND6 proteins in ram sperm. The images revealed that the presence of bands representing MT-ND1 and MT-ND6 proteins in ram sperm (Figure 6A). Notably, the levels of expression of MT-ND1 and MT-ND6 proteins in sperm treated with the 10 nM and 100 nM MA-5 treatments were significantly higher than in the control, and the 10 nM MA-5 treatment had the highest value among the treatments (Figure 6A–C and Supplementary Figure S2, *p* < 0.05).

## 4. Discussion

A low storage temperature causes severe damage to sperm due to dramatic temperature changes and exposure to osmotic and toxic stresses resulting from the formation and dissolution of ice in the intracellular and extracellular environments [26]. These changes impair sperm transport and survival in the female reproductive tract, consequently reducing fertility in domestic species [27]. 

ATP synthesis in the mitochondria is the source of over 90% of energy in eukaryotic cells, and it is produced through oxidative phosphorylation (OXPHOS) with ROS as by-products [28,29]. While ROS are required for tyrosine phosphorylation in sperm and the sperm–egg interaction, the accumulation of ROS leads to structural and functional damage to components of the sperm cell, and this affects sperm motility [30]. Overall, the lower level of mitochondrial activity that occurs during cold storage is closely associated with lower viability, the concentration of spermatozoa, and sperm motility [31,32]. In this study, we reported for the first time that the addition of 10 nM MA-5 to a semen extender improved sperm kinetic parameters and preserved the integrity of the plasma membrane and acrosome of ram sperm after exposure to low temperatures.

MA-5, a derivative of the plant hormone indole-3-acetic acid, acts as an accelerator, enhancing ATP synthesis [19,21], and it also has a cell-protective effect on microglia [33]. In this study, the addition of 10 nM MA-5 to a semen extender significantly improved sperm motility. Furthermore, the mitochondrial membrane potential plays a crucial role in ATP synthesis [34]. This process facilitates the creation of a localized proton gradient, leading to ATP production while mitigating mitochondrial ROS generation. Our results also show an increase in semen ATP contents with the supplementation of 10 nM MA-5 to the extender. This suggests that MA-5 has discernible influence on mitochondrial OXPHOS or membrane potential. 

The sperm membrane is a structure that undergoes reorganization during capacitation, but cold shock reduces membrane permeability to water and solutes and injures acrosomal membranes [35]. The plasma membrane and acrosome integrity of sperm decreased with storage at 4 °C for 48 h in the control, leading to a corresponding reduction in sperm motility. The relationship between ROS accumulation and mitochondria DNA damage have been reported by Baumber et al., who stated that mitochondrial DNA damage occurs as a result of ROS damage to the mitochondrial membrane, causing a change in its potential difference with corresponding decreases in ATP generation and sperm motility [36]. Our result demonstrated that the addition of 10 nM MA-5 to the sperm extender preserved the integrity of the plasma membrane and acrosome of sperm after exposure to low temperatures Mitochonic acid 5 interventions in mitochondrial diseases have improved the survival of fibroblasts by increasing ATP contents and enhancing plasma membrane function [19]. 

Mitochondrial proteins play a vital role in maintaining cristae junctions, contributing to the preservation of mitochondrial structure and function [37]. Ram sperm mitochondria are influenced by mitochondrial energy proteins, impacting ATP contents. It is well-established that sperm cells necessitate ATP for flagellar movement, and this reliance is contingent on OXPHOS to fulfill their energy requirement [38]. In addition, the MT-ND1 and MT-ND6 genes are part of complex 1 [39]. Complex I plays a key role in OXPHOS by receiving electrons from NADH to complete the ATP generation process [40]. In this study, the 10 nM MA-5 treatment was observed to increase the presence of mitochondrial proteins (MT-ND1 and MT-ND6), indicating potential effects on enhancing sperm mitochondrial functions to withstand liquid storage at 4 °C. This may be responsible for the improvement in sperm motility and progressive motility observed in the 10 nM MA-5 treatment compared to other treatments. Thus, this indicates that the influence of MA-5 on the *MT-ND1* and *MT-ND6* genes may affect the activity of complex I, resulting in an advantage in energy production without elevating ROS levels. Interestingly, the 10 nM concentration of MA-5 also generated the lowest ROS levels in this study. This agrees with the findings of Maranzana et al., who stated that MA-5 produces ATP without generating excesses mitochondrial ROS by mitochondrial cristae modification and supercomplex formation [41].

## 5. Conclusions

**Figure 7:**
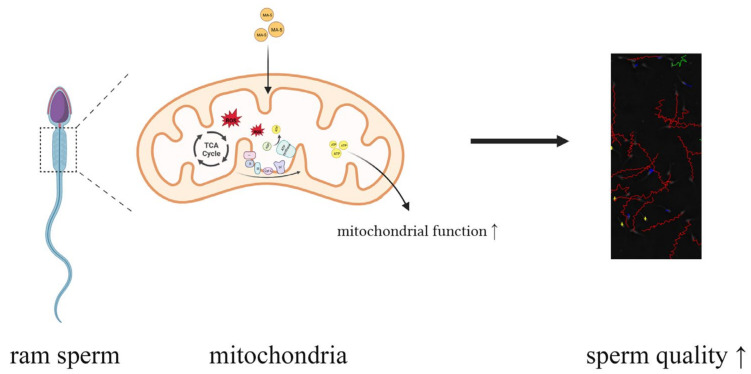
The mechanism of MA-5 in maintaining sperm motility. MA-5 protected sperm from ROS damage and promoted mitochondrial gene expression and protein synthesis to keep sperm generating an appropriate amount of ATP to maintain ram sperm motility.MA-5: mitochonic acid 5. TCA Cycte: tricarboxylic acid cycle; ATP: adenosine 5′-triphosphate; ROS reactive oxygen species; I: NADH dehydrogenase; II: succinate dehydrogenase; III: CoQH2-cytochrome c reductase; IV: cytochrome c oxidase.

As shown in Figure 7, a low storage temperature limits mitochondrial membrane function; however, the supplementation of MA-5 at 10 nM enhances mitochondrial function without excessive the ROS generation that occurs during high ATP production through OXPHOS, thereby, improving ATP production and membrane and acrosomal integrity (Figure 7). Thus, our results suggest that MA-5 may serve a valuable key role in improving sperm function by increasing high sperm mitochondria function, modulating ROS homeostasis, and mediating an efficient energy metabolism process in sperm mitochondria.

## Figures and Tables

**Table 1:** Effect of MA-5 on ram sperm in liquid storage at 4 °C by CASA.

Different Concentrations of MA-5 (μM)						
Sperm Parameters	Time (h)	0	0.1	1	10	100
No progression (%)	12	39.01 ± 1.82 ^a^	30.3 ± 0.92 ^b^	33.58 ± 1.59 ^ab^	25.05 ± 0.71 ^c^	34.31 ± 1.71 ^ab^
	24	59.98 ± 1.04 ^a^	61.55 ± 1.10 ^a^	59.44 ± 1.09 ^a^	49.07 ± 0.61 ^c^	55.59 ± 0.34 ^b^
	48	61.73 ± 2.21 ^a^	57.99 ± 3.7 ^a^	49.87 ± 7.91 ^c^	41.32 ± 11.13 ^d^	54.95 ± 1.79 ^b^
VSL (μm/s)	12	42.1 ± 1.39 ^b^	43.42 ± 1.29 ^b^	42.12 ± 3.28 ^b^	52.13 ± 1.04 ^a^	43.22 ± 1.16 ^b^
	24	23.34 ± 0.90 ^c^	22.58 ± 2.58 ^d^	22.56 ± 1.13 ^d^	33.63 ± 0.88 ^a^	30.53 ± 0.52 ^b^
	48	29.5 ± 4.3 ^b^	31.05 ± 3.82 ^b^	39.8 ± 2.36 ^a^	48.65 ± 4.42 ^a^	39.8 ± 1.81 ^a^
VCL (μm/s)	12	73.69 ± 1.23 ^b^	70.32 ± 1.04 ^b^	74.11 ± 3.30 ^b^	81.18 ± 1.70 ^a^	73.97 ± 1.00 ^a^
	24	62.26 ± 1.23 ^c^	62.77 ± 0.59 ^c^	65.67 ± 1.97 ^c^	81.99 ± 7.30 ^a^	75.83 ± 2.21 ^b^
	48	60.97 ± 3.19 ^b^	62.67 ± 1.82 ^b^	72.07 ± 1.76 ^a^	70.2 ± 2.45 ^a^	72.08 ± 3.95 ^a^
VAP (μm/s)	12	48.87 ± 1.30 ^c^	45.50 ± 2.12 ^c^	49.29 ± 1.30 ^b^	56.37 ± 2.43 ^a^	49.15 ± 1.23 ^bc^
	24	37.44 ± 0.2 ^c^	37.95 ± 0.53 ^c^	48.41 ± 0.62 ^b^	57.17 ± 0.72 ^a^	51.06 ± 1.99 ^b^
	48	36.15 ± 0.88 ^b^	37.83 ± 4.35 ^a^	39.69 ± 0.42 ^a^	45.15 ± 1.15 ^a^	47.25 ± 0.9 ^a^
LIN (%)	12	53.09 ± 1.37 ^b^	49.72 ± 1.19 ^b^	53.51 ± 2.32 ^b^	60.58 ± 1.78 ^a^	53.37 ± 0.9 ^b^
	24	41.66 ± 1.3 ^d^	42.17 ± 4.43 ^d^	52.63 ± 3.10 ^c^	61.3 ± 0.72 ^a^	55.28 ± 1.07 ^b^
	48	40.37 ± 4.08 ^b^	42.01 ± 2.52 ^b^	43.91 ± 7.2 ^ab^	49.62 ± 1.15 ^a^	51.42 ± 4.47 ^a^
STR (%)	12	78.50 ± 1.15 ^bc^	75.13 ± 5.23 ^c^	78.92 ± 2.29 ^b^	85.99 ± 1.24 ^a^	78.78 ± 1.21 ^b^
	24	67.07 ± 0.40 ^e^	67.58 ± 0.36 ^d^	78.04 ± 0.51 ^c^	86.80 ± 7.40 ^a^	80.69 ± 4.49 ^b^
	48	65.78 ± 3.29 ^d^	67.46 ± 1.82 ^c^	69.32 ± 0.52 ^b^	75.04 ± 0.11 ^a^	76.89 ± 4.54 ^a^
WOB (%)	12	60.23 ± 1.30 ^bc^	56.86 ± 1.17 ^c^	60.65 ± 3.41 ^b^	67.72 ± 1.75 ^a^	60.51 ± 0.89 ^b^
	24	48.80 ± 3.10 ^c^	49.31 ± 0.47 ^c^	59.77 ± 3.40 ^b^	68.10 ± 2.57 ^a^	62.40 ± 0.7 ^b^
	48	47.51 ± 7.47 ^c^	49.19 ± 0.52 ^c^	51.05 ± 3.4 ^b^	56.71 ± 1.57 ^a^	58.62 ± 0.97 ^a^

Values are presented as means ± standard deviation (SDs). Parameters include VCL (curvilinear velocity), VSL (straight-line velocity), VAP (average path velocity), STR (straightness, calculated as VSL/VAP), LIN (linearity, calculated as VSL/VCL), and WOB (wobble, calculated as VAP/VCL). Significant differences (*p* < 0.05), indicated by different letters within a line, were determined using a one-way ANOVA and Dunnett’s test, comparing the results to the medium without MA-5.

## Data Availability

The data presented in this study are available in the article.

## Supplementary Materials

The following supporting information can be downloaded at: https://www.mdpi.com/article/10.3390/ani14030368/s1, Figure S1: A: Sperm with bright green fluorescence are considered intact (white arrow), while sperm with red fluorescence indicate plasma membrane damage (red arrow). B: sperm showing bright green fluorescence is considered complete (white arrows), while sperm with no green or speckled green fluorescence indicates acrosome damage (red arrows); Figure S2: Effect of different concentrations of mitochonic acid 5 on the expression of mitochondria proteins (MT-ND1 and MT-ND6) in ram sperm store.
