# Supplementary material for: Mitochonic Acid 5 Increases Ram Sperm Quality by Improving Mitochondrial Function during Storage at 4 °C
Source: Animals (Basel). 2024 Jan 23;14(3):368. doi: 10.3390/ani14030368 (PMC10854625; doi:10.3390/ani14030368)
Supplement: Supplementary file 1 [file animals-14-00368-s001.zip › animals-2832144-supplementary.pdf]

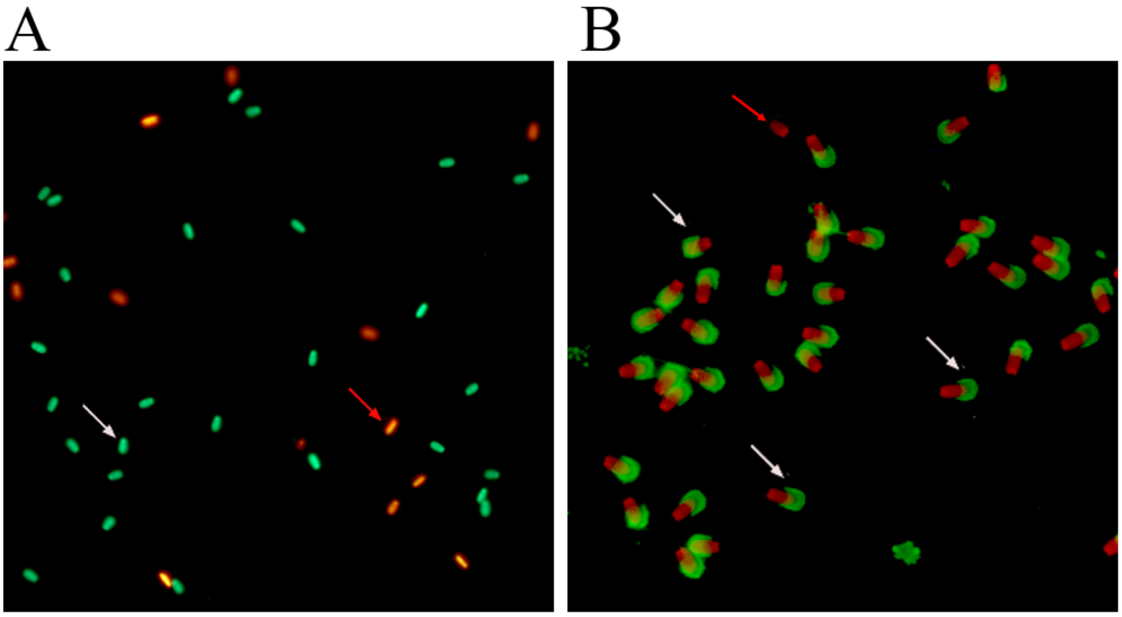

Figure S1. A： Sperm with bright green fluorescence are considered intact (white arrow), while sperm with red fluorescence indicate plasma membrane damage (red arrow). B: sperm showing bright green fluorescence is considered complete (white arrows), while sperm with no green or speckled green fluorescence indicates acrosome damage (red arrows).

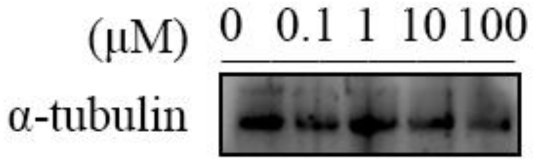
(μM)
0 0.1 1 10 100
α-tubulin

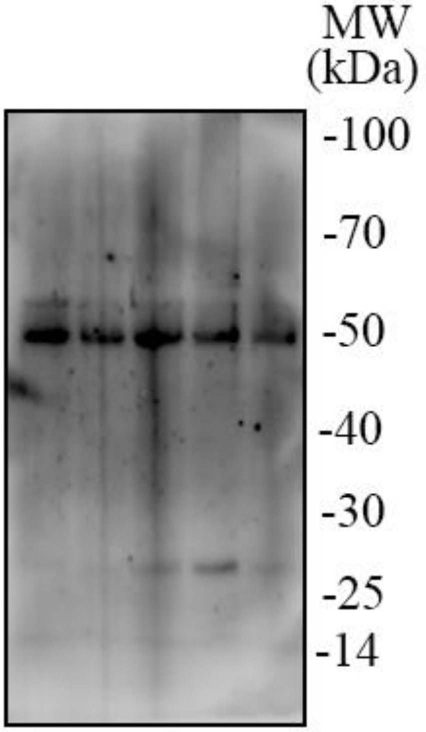
MW (kDa)
-100
-70
-50
-40
-30
-25
-14

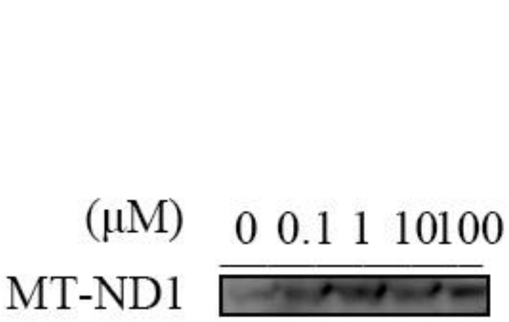
(μM)
0 0.1 1 10100
MT-ND1

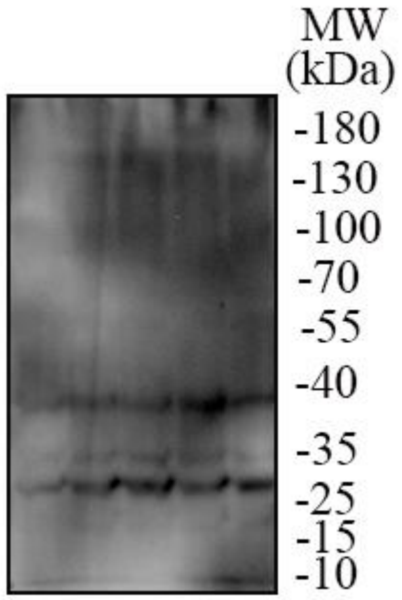
MW (kDa)
-180
-130
-100
-70
-55
-40
-35
-25
-15
-10

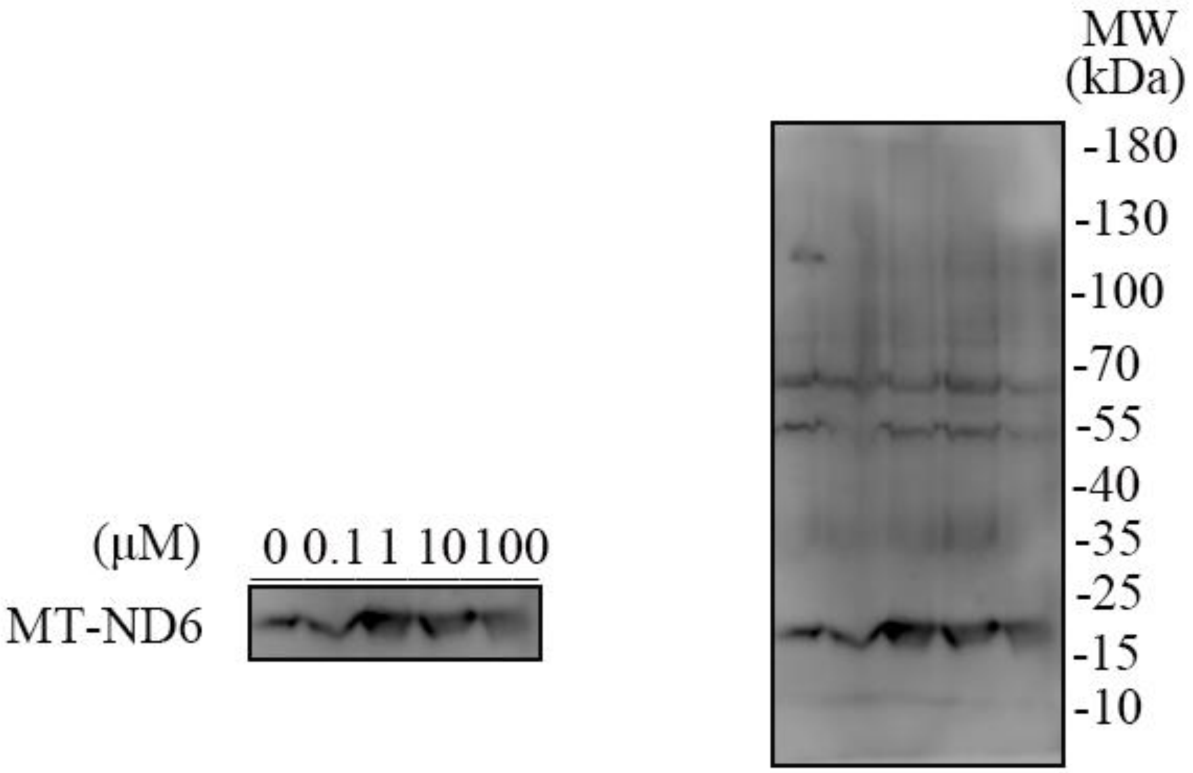

Figure S2. Effect of different concentrations of mitochonic acid 5 on the expression of mitochondria proteins (MT-ND1 and MT-ND6) in ram sperm.
